# Microbial Assessment of Tomatoes (*Lycopersicon esculentum*) Sold at Some Central Markets in Ghana

**DOI:** 10.1155/2018/6743826

**Published:** 2018-11-29

**Authors:** Forson Akua Obeng, Pokuaa Belinda Gyasi, Michael Olu-Taiwo, F. Patrick Ayeh-kumi

**Affiliations:** ^1^Department of Medical Laboratory Science, School of Biomedical and Allied Health Sciences, College of Health Sciences, University of Ghana, Legon, Accra, Ghana; ^2^Department of Medical Microbiology, School of Biomedical and Allied Health Sciences, College of Health Sciences, University of Ghana, Legon, Accra, Ghana

## Abstract

**Background:**

Tomato (*Lycopersicon esculentum*) has a high water content which predisposes it to spoilage by pathogenic bacteria that can pose significant health threats to consumers.

**Aim:**

The study aimed to determine the various pathogenic bacteria associated with tomatoes sold in some central markets in the Accra Metropolis.

**Method:**

A total of 120 tomatoes were sampled, out of which 60 fresh, firm, undamaged tomatoes and 60 spoilt tomatoes were analysed. Cut portions of the fresh and spoilt tomatoes were swabbed with sterile swabs and cultured on Blood agar, Nutrient agar, and MacConkey agar. The antibiogram of bacterial isolates was determined by Kirby-Bauer disc-diffusion method.

**Results:**

Out of the 120 tomatoes analysed, a total of 66 bacterial isolates were recovered, 68.2% were associated with spoilt tomatoes, and 31.8% were from fresh tomatoes.* Klebsiella *sp. (34.8%),* Enterobacte*r sp. (24.2%), and* Citrobacter* sp. (7.6%) were the predominant bacteria isolated. Agbogbloshie market (36.4%) had both fresh (18.2%) and spoilt (18.2%) tomatoes contaminated, whilst Makola market (31.8%) had a higher spoilt (30.3%) tomatoes contaminated. Although none of the isolates expressed resistance to ciprofloxacin, resistance was found for ampicillin (63.1%), tetracycline (60.1%), and cefuroxime (59.1%).

**Conclusion:**

Varying levels of antibiotic resistance bacteria amongst tomatoes sold at various markets were found. Contamination might have been caused by poor sanitation, improper handling or transportation from the farms to the markets. The presence of antibiotic resistance bacteria amongst tomatoes raises concern on public health risks associated with the consumption of fresh tomatoes.

## 1. Introduction

Tomato (*Lycopersicon esculentum)* is a perishable vegetable widely cultivated and consumed worldwide [1, 2]. It is rich in nutrients, vitamins, dietary fibres, and phytochemicals [3–5]. It is known to be a very profitable crop that provides high returns for small scale farmers in most developing countries [6]. Due to its nutritive value, taste, affordability, and accessibility, there has been an increase in demand by consumers [7]. However, isolation and identification of microorganisms that are associated with spoilage of tomatoes have gained some research focus [8].

In most developing countries, microbial infestation of tomatoes can occur during the harvesting period, postharvesting, handling, storage, transportation, and processing by customers [9, 10]. Baiyewu et al. [11] have also reported that another means of bacterial contamination is by exposing them on benches and baskets in the open markets for customers. The proliferation of bacteria more especially in damaged tomatoes could be considered to be more harmful when such contaminated tomatoes are consumed in improperly cooked food [2].

Some studies have been carried out on bacteria associated with tomatoes and tomato products in some countries. A study carried out by Ajayi [12] in the United State has revealed that* Clostridium *sp*., Staphylococcus *sp., and* Bacillus *sp. were predominant bacteria isolated from both canned and raw tomatoes. In India, a study carried out on tomato puree revealed the presence of* Klebsiella *sp*., Proteus mirabilis, Vibrio *sp*., and Pseudomonas *sp. [13]. In Nigeria, Wogu and Ofuase [14] isolated* Bacillus subtils*,* Klebsiella aerogenes*,* Pseudomonas aeruginosa*,* Salmonella typhi*,* Proteus mirabilis,* and* Staphylococcus aureus* from spoilt tomatoes in Benin City. A similar study also revealed high levels of* Staphylococcus* sp. (22.5%),* Bacillus* sp. (20%), and* Escherichia coli* (15%) in Lagos State, Nigeria [15]. In Ghana, limited information is available on the types of pathogenic bacteria associated with tomatoes sold in markets in Accra, Ghana. This study aimed to isolate and identify pathogenic bacterial agents associated with two different grades of raw tomatoes (fresh and spoilt) sold in three central markets in Accra.

## 2. Methodology

### 2.1. Study Area

#### 2.1.1. Sample Collection

An experimental study was carried out by randomly purchasing tomatoes from different sellers at three different markets (Agbogbloshie, Makola, and Kaneshie) in Accra. These markets were selected because they are major markets in the region where tomatoes are sold at cheaper prices to consumers.

**Figure 1:**
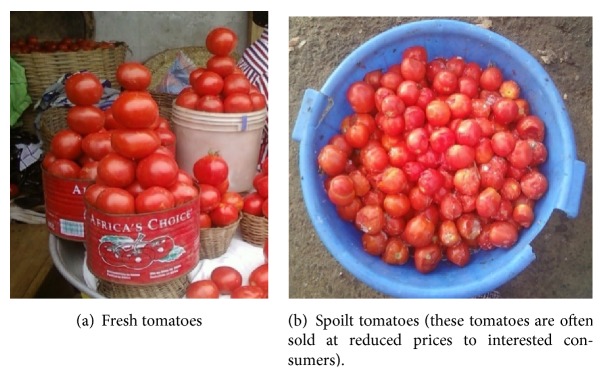
Pictures of some displayed tomatoes in markets.

A total of 120 tomatoes were randomly purchased from the three markets in Accra. In each market, 20 fresh (Figure 1(a), firm and undamaged) and 20 spoilt (Figure 1(b), damaged and spoilt) tomatoes were purchased. Ten sellers were selected from each market and two fresh and spoilt tomatoes each were purchased. Samples were separately packaged into different sterile containers, labelled, and transported to the laboratory immediately for bacteriological analysis. The elapsed time between sample collection and analysis did not exceed 2 hrs.

#### 2.1.2. Laboratory Analysis

Tomatoes was analysed using Ugwu et al.'s [16] method. Briefly, fresh tomatoes were individually washed with sterile water before the tomatoes were cut into two equal halves (this was carried out because this is a common practice by consumers before food preparation). Aseptically, a sterile swab stick was used to swab the cut interior of the tomatoes. The swabs were then streaked onto Blood agar (Oxoid, UK), Nutrient agar (Oxoid, UK), and MacConkey agar (Oxoid, UK) and incubated at 37°C for 18-24 hours.

For the spoilt tomatoes, a sterile swab was used to take samples from the spoilt portion of the tomatoes and this was streaked onto Blood agar, Nutrient agar, and MacConkey agar before incubation at 37°C for 18-24 hrs.

#### 2.1.3. Identification of Organisms

After incubation, the colonies of the different culture media were examined and recorded based on the shape, colour, border, texture, and general appearance of individual bacterial colonies on each plate, and single representative colony was Gram stained [17]. Gram staining was done to reveal the characteristic group and arrangement of the cells. Biochemical tests (indole test, methyl red test, Voges-Proskauer (VP) test, citrate test, oxidase test, coagulase test, and sugar fermentation test) were then carried out for the identification of bacterial isolates.

#### 2.1.4. Susceptibility Testing

Antibiogram of all bacterial isolates were carried out using Kirby-Bauer disk diffusion method [18] to ampicillin (10*μ*g), chloramphenicol (10*μ*g), cefotaxime (30*μ*g), ceftriaxone (30*μ*g), gentamicin (10*μ*g), cefuroxime (30*μ*g), meropenem (10*μ*g), amikacin (30*μ*g), cotrimoxazole (25*μ*g), ciprofloxacin (5*μ*g), and tetracycline (10*μ*g). These antibiotics are commonly used for the treatment of bacterial infections in the general populace [19].

Briefly, stored isolates were subcultured onto horse blood agar plates (37°C, 18 hrs.) and individual colonies were suspended in saline to a turbidity equivalent to 0.5 McFarland standard. The suspensions obtained were then streaked on Mueller-Hinton agar plate (Oxoid, UK) using sterile swab sticks. The paper discs were gently but firmly placed on the inoculated plates before the plates were incubated at 37°C for 18-24 hrs. After incubation, zones of inhibition were measured and interpreted according to Clinical and Laboratory Standard Institute [18], whilst other break points were sourced from EUCAST [20] (European Committee on Antimicrobial Susceptibility Testing).

The reference strains used for the determination of the MIC values were* E. faecalis* ATCC 29212 and* Staphylococcus aureus* ATCC 29213.

#### 2.1.5. Data Analysis

The quantitative data generated from the study was coded and fed into Microsoft Excel and analysed using GraphPad Prism software, version 6. In all cases, P values less than 0.05 were considered statistically significant. Fisher exact test was carried out to test the significance of prevalence of bacteria in the various markets.

## 3. Results

### 3.1. Distribution of the Bacteria in Three Central Markets in Accra

Out of the 120 tomatoes purchased from the three markets (Agbogbloshie, Kaneshie, and Makola), eleven different bacteria were isolated (Table 1). They were* Bacillus* sp.,* Enterobacter* sp.,* Citrobacter* sp.,* Klebsiella* sp.,* Shigella* sp.,* Proteus mirabilis*,* Klebsiella oxytoca*,* Enterobacter cloacae*,* Citrobacter koseri,* and* Klebsiella pneumoniae*. Tomatoes sampled from Agbogbloshie market (36.4%) were the most contaminated in both fresh (18.2%) and spoilt (18.2%) tomatoes with similar prevalence of bacterial contamination. In contrast, tomatoes sampled from Kaneshie (31.8%) had few fresh (12.1%) and spoilt (19.7%) tomatoes contaminated, and Makola (31.8%) market had higher spoilt (30.3%) tomatoes contaminated.

Significant difference was found between Kaneshie and Makola markets (p = 0.0021) and Agbogbloshie and Makola markets (p = < 0.0001), when the prevalence of isolated bacteria was evaluated. However, no significant difference was found between Agbogbloshie and Kaneshie markets (p = 0.5503).

### 3.2. Occurrence of Bacteria in Sampled Fresh and Spoilt Tomatoes

Table 2 shows the percentage distribution of isolates in fresh and spoilt tomatoes purchased from the different markets. Out of a total of 66 isolates isolated, 68.2% were associated with spoilt tomatoes, whilst 31.8% were on fresh tomatoes.

*Klebsiella *sp. (34.8%) was the predominant isolates with 10 of the fresh and 13 spoilt tomatoes being positive.* Enterobacter *sp. (24.2%) followed with 7 of the fresh tomatoes and 9 of the spoilt tomatoes. Whilst* Klebsiella oxytoca* (12.1%) was next with 1 isolate in the fresh tomatoes and 7 in the spoilt ones,* Citrobacter* sp. (7.6%) was found in only two fresh tomatoes. Except for* Enterobacter cloaca *which was found in 1 of the fresh and 2 of the spoilt tomatoes,* Bacillus *sp. (3%) was found in 2 of the fresh tomatoes.* Shigella *sp.,* Proteus aeruginosa*,* Proteus mirabilis,* and* Citrobacter koseri *were not isolated from any of the fresh tomatoes but were found in 2 of the spoilt ones, respectively. Finally, the one with the least occurrence was* Klebsiella pneumoniae *which was found in only one of the spoilt tomatoes.

### 3.3. Antibiogram

**Figure 2:**
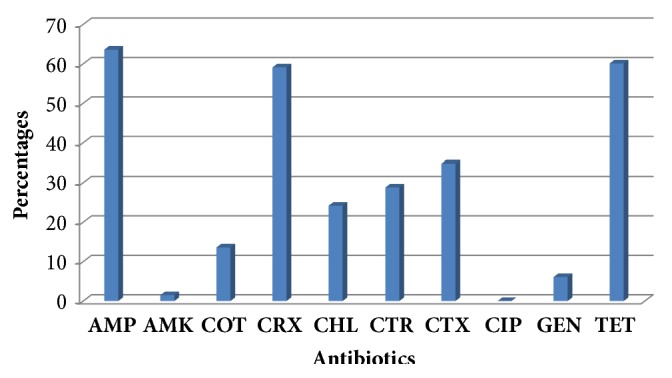
Percentage distribution of resistance pattern of bacteria isolated from tomatoes.** AMP**=Ampicillin,** AMK**=Amikacin,** COT**=Cotrimoxazole,** CRX**=Cefuroxime,** CHL**=Chloramphenicol,** CTR**=Ceftriaxone,** CTX**=Cefotaxime,** CIP**=Ciprofloxacin,** GEN**=Gentamicin, and** TET**=Tetracycline.

The resistance levels of ten tested antibiotics for all the bacterial isolates are presented in Figure 2. The results for the different bacterial species have been combined to enable comparison. Resistance to ampicillin, amikacin, cotrimoxazole, cefuroxime, chloramphenicol, ceftriaxone, cefotaxime, ciprofloxacin, meropenem, and tetracycline was found in all grades of tomatoes.

In total, resistance to ampicillin (63.6%), cefuroxime (59.1%), and tetracycline (60.1%) was found to be the highest (Figure 2). However, a slightly lower prevalence was found for cefotaxime, (34.8%) and ampicillin (37.9%), ceftriaxone (28.8%), chloramphenicol (24.2%), and cotrimoxazole (13.6%). Whilst a very low resistance level was found for gentamicin (6.1%) and amikacin (1.5%), none of the isolates was resistant to ciprofloxacin.

Furthermore, most of the* Citrobacter* sp.,* Klebsiella* sp., and* Enterobacter* sp. were multiresistant to ampicillin, tetracycline, or cefotaxime (Table 3). However, a few* Enterobacter *sp. (2 isolates),* Citrobacter Koseri* (1 isolate), and* Klebsiella* sp. (1 isolate) were resistant to more than five different antibiotics.

## 4. Discussion

The present study reports for the first time varying prevalence of resistant bacteria in sampled tomatoes purchased from the different sellers in selected central markets (Agbogbloshie, Kaneshie, and Makola markets) in Accra, Ghana. In contrast to Kaneshie (34.78%) and Makola (21.74%) markets, Agbogbloshie (43.48%) recorded the highest level of contamination. This suggests that Agbogbloshie market is not as hygienic as compared to Makola and Kaneshie markets. The presence of bacteria in the fresh tomatoes bought from these markets may be because they were improperly handled during the sellers' attempts to arrange them for sales [21]. The varying differences in contamination from the three markets could also be as a result of differences in the sources of farm products or wholesale points where the market sellers bought their tomatoes from [22].

In this study,* Klebsiella* sp. (34.8%) was the prevalent bacteria isolated from both spoilt tomatoes (19.7%) and fresh tomatoes (15.2%). This is in contrast to Ugwu et al.'s [16] study in Nigeria which reported isolation rate of 8.9% in only spoilt tomatoes and Wogu and Ofuase [16] study from Benin City, Nigeria, with a total isolation rate of 1.6% for* Klebsiella* sp. Whilst in Spain, Falomir et al. [23] have isolated* Klebsiella pneumoniae* and* Klebsiella oxytoca* in their work on fresh vegetables; the varying prevalence of* Klebsiella* isolates in the different countries could be because of varying human activities associated with postharvest practices before the tomatoes are displayed for sale in the different countries. In addition,* Klebsiella *sp. are ubiquitous organisms that can be found in the environment, animals, and humans [24, 25]. The bacteria could have gained access to the tomatoes during postharvest period involving poor transportation and storage facilities on the field with stomata that have openings, cracks, or surface injuries as reported by Lemma et al. [6].

In this study, prevalence for* Enterobacter *sp. (24.2%) was found to be slightly higher than the 21.4% reported by Adebayo-Tayo et al. [26], in Uyo Metropolis, Nigeria. Their presence in tomatoes may be due to handling practices by the vendors [27]. The high incidence of* Klebsiella *sp. and* Enterobacter* sp. is an indication of human contact, since improper handling of tomatoes during market days may have introduced these organisms into the tomatoes [28].

The presence of* Enterobacter *sp*., Citrobacter *sp*., Proteus mirabilis,* and* Bacillus *sp. in this study is in conformity with Ogundipe et al. [15], which isolated similar bacteria with percentages of 12.5%, 2.5%, 2.5%, and 20.0%, respectively, from tomatoes in Lagos State, Nigeria. However,* Citrobacter* sp. was found to be 7.6% in this study which is lower than the 30% reported by Mahamud et al. [29], in Northern Nigeria on tomatoes.* Citrobacter* sp. and* Citrobacter koseri *are often present in soils, water, or wastewater and can cause infections in the urinary tract and sepsis in humans. Their presence in the tomatoes could have been introduced from the soil in which the tomatoes were planted or as a result of irrigation with contaminated wastewater. The percentage of* Bacillus* sp. isolated in this study was 3.0%, which is lower than 59.1% reported by Wogu and Ofuase [14] in previous study on tomatoes in Benin City, Nigeria. The difference in prevalences may be associated with varying incidence of* Bacillus* sp. spores in the environment [30]. In addition,* Bacillus *sp. are resistant to killing by high temperatures of the sun's ultraviolet rays because of the endospores, hence their bacterial load in the tomatoes. In this study,* Proteus mirabilis* isolated confirms with a previous work done by Garg* et al.* [14] in India with tomatoes.* Proteus mirabilis* is an opportunistic pathogen in the normal intestinal flora and they may be associated with community-acquired infection [31]. It is widely distributed in contaminated soil and water in the natural environment and can easily find its way into foodstuffs which are not well handled.

The presence of* Shigella* sp. (3.0%) in only the spoilt tomatoes is an indication that the tomatoes may have been exposed to faecal-contaminated water or manure during cultivation [32].* Shigella *sp. isolated in this study is in contrast to Adebayo-Tayo et al.'s [26] study in Uyo Metropolis, Nigeria, which reported no* Shigella* sp*. Shigella *sp. can contaminate tomatoes when they are exposed to faecal-contaminated water or improper hygiene prior to handling of the tomatoes [33].

Furthermore, most of the isolates were susceptible to gentamicin (93.9%) and amikacin (98.5%), but none of the isolates expressed resistance to ciprofloxacin. However, high resistance was observed for ampicillin (63.1%), tetracycline (60.1%), and cefuroxime (59.1%). The varying antibiotic prevalence has been previously reported by Wogu and Ofuase [14] in a previous study on tomatoes in Benin City, Nigeria. The difference in resistance may be associated with varying functional groups of antibiotics and bacterial species. The presence of bacteria with antibiotic resistance associated with tomatoes sampled in this study highlights the potential risk of tomatoes to consumers.

## 5. Conclusion

The different bacterial species identified in this study suggest that bacteria contamination on tomatoes can be a potential risk to consumers. Such contamination can lead to food poisoning and food-borne illnesses. As a result, efforts should be made by consumers to discourage purchasing spoilt tomatoes from local markets in Accra as they can predispose vendors and the general public to infection.

## 6. Recommendations

To prevent any outbreak of diseases, tomatoes should be thoroughly washed with clean water and disinfected before use or properly cooked before consumption.

If possible, consumption of raw or partially cooked tomatoes in the form of sandwich or salads should be avoided, since it can predispose consumers to bacterial infections.

The environment in which the tomatoes are sold should also be kept clean since most of the bacteria isolated are associated with dirty environment. Tomato farmers as well as other vegetable farmers should be advised to avoid the use of contaminated wastewater for irrigation during cultivation.

## 7. Limitations

The current study had certain limitations; only a few tomatoes were sampled due to poor cooperation of vendors and some farmers (when approached). The results of this study cannot be generalized to all tomatoes sold in Ghana as variations in seasons, handling, harvesting, and postharvesting practices are different in many regions in Ghana. Other limitations include lack of baseline data on handling practices of vendors and farmers and as a result it is impossible to determine the source of the microorganisms detected in this study. A larger survey that will incorporate farms, farmers, and vendors to determine the sources of contamination and other variables is planned pending appropriate funding.

## Figures and Tables

**Table 1:** Distribution of the bacteria isolates in different grades of tomatoes from three major markets in Accra.

	**Fresh tomatoes**			
	Markets (no. of sampled tomatoes)			
**Isolates**	**Agbogbloshie (n = 20)**	**Kaneshie (n = 20)**	**Makola (n = 20 )**	**Total (**%**)**
***Bacillus *sp.**	0	2	0	
***Citrobacter *sp.**	0	0	0	
***Citrobacter koseri***	0	0	0	
***Enterobacter *sp.**	6	1	0	
***Enterobacter cloacae***	0	1	0	
***Klebsiella *sp.**	5	4	1	
***Klebsiella oxytoca***	1	0	0	
***Klebsiella pneumoniae***	0	0	0	
***Proteus mirabilis***	0	0	0	
***P. aeruginos*a**	0	0	0	
***Shigella *sp.**	0	0	0	

**Total (**%**)**	**12 (18.2)**	**8 (12.1)**	**1 (1.5)**	**21 (31.8)**

	**Spoilt tomatoes**			
	Markets (no. of sampled tomatoes)			
**Isolates**	**Agbogbloshie (n = 20)**	**Kaneshie (n = 20)**	**Makola (n = 20 )**	

***Bacillus *sp.**	0	0	0	
***Citrobacter *sp.**	0	2	3	
***Citrobacter koseri***	0	0	2	
***Enterobacter *sp.**	3	4	2	
***Enterobacter cloacae***	2	0	0	
***Klebsiella *sp.**	2	3	8	
***Klebsiella oxytoca***	3	2	2	
***Klebsiella pneumoniae***	1	0	0	
***Proteus mirabilis***	0	1	1	
***P. aeruginosa***	0	0	2	
***Shigella *sp.**	1	1	0	

**Total**	**12 (18.2)**	**13 (19.7)**	**20 (30.3)**	**45 (68.2)**

**Total no. of isolates from fresh and spoilt tomatoes**	**24(36.4)**	**21(31.8)**	**21(31.8)**	**66(100**%**)**

**Table 2:** Total distribution of bacteria isolates in fresh and spoilt tomatoes.

	**Grade of Tomatoes**		
**Isolates**	**Fresh tomatoes**	**Spoilt tomatoes**	**Total (**%**)**
***Bacillus *species**	2	0	2 (3.0)
***Citrobacter *species**	0	5	5 (7.6)
***Citrobacter koseri***	0	2	2 (3.0)
***Enterobacter *species**	7	9	16 (24.2)
***Enterobacter cloacae***	1	2	3 (4.5)
***Klebsiella *species**	10	13	23 (34.8)
***Klebsiella oxytoca***	1	7	8 (12.1)(
***Klebsiella pneumoniae***	0	1	1 (1.5)
***Proteus mirabilis***	0	2	2 (3.0)
***P. aeruginosa***	0	2	2 (3.0)
***Shigella *species**	0	2	2 (3.0)

**Total **	**21 (31.8) **	**45 (68.2)**	**66 (100)**

**Table 3:** Distribution of Multidrug resistant (MDR) bacteria isolates in fresh and spoilt tomatoes.

**Isolates (total no. isolated)**	**No. of MDR isolates **	**Resistant pattern**	%** of MDR**
***Citrobacter *species (5)**	1	AMP-TET-CRX	40
	1	AMP-CRX-CTD	
	2	AMP-TET-CRX-CTX	
***Citrobacter koseri *(5)**	1	TET-GEN-CRX-CHL-CTX	40
	1	AMP-TET-COT-CRX-CHL-CTX	
***Enterobacter *sp. (16)**	1	CRX-CTR-CTX	25
	1	AMP-CRX-CTR-CTX	
	1	AMP-TET-GEN-CRX-CTR-CTX	
	1	AMP-TET-COT-CRX-CHL-CTX	
***Enterobacter cloacae* (3)**	1	AMP-TET-CRX-CTX	33
***Klebsiella *sp. (10)**	1	TET-CRX-CTR-CTX	40
	1	AMP-CRX-CTR-CTX	
	1	AMP-TET-CRX-CHL-CTR-CTX	
	1	AMP-CRX-CTX	
***Klebsiella oxytoca *(8)**	1	AMP-TET-CRX	50
	1	AMP-CRX-CTX	
	2	AMP-TET-CRX-CTX	
***Klebsiella pneumonia *(1)**	1	AMP-CRX-CTX	100
***Proteus mirabilis *(2)**	1	AMP-CRX-CTR-CTX	50

TET= Tetracycline, COT= Cotrimoxazole, GEN= Gentamicin, CRX= Cefuroxime, CHL= Chloramphenicol, CTX= Cefotaxime, CTR= Ceftriaxone, MEM= Meropenem, AMK= Amikacin, CIP= Ciprofloxacin, and AMP= Ampicillin.

## Data Availability

The datasets used and/or analysed during the current study are available from the corresponding author upon reasonable request.
